# Chemical weathering and CO_2_ consumption rates of rocks in the Bishuiyan subterranean basin of Guangxi, China

**DOI:** 10.1038/s41598-020-68572-4

**Published:** 2020-07-15

**Authors:** Pingping Jiang, Guo Yu, Qiang Zhang, Yane Zou, Qingjia Tang, Zhiqiang Kang, Pen Sytharith, He Xiao

**Affiliations:** 1 College of Environmental Science and Engineering, Guilin University of Technology, Guilin, 541004 China; 2 Key Laboratory of Karst Dynamics, Ministry of Natural Resources and Guangxi Zhuang Autonomous Region, Institute of Karst Geology, Chinese Academy of Geological Sciences, Guilin, 541004 Guangxi China; 3 School of Environmental Studies, China University of Geosciences, Wuhan, 430074 China; 4 School of Water Resources and Environment, China University of Geosciences, Beijing, 100083 China; 5 Guangxi Geological Survey, Nanning, 530023 China; 6 Faculty of Hydrology and Water Resources Engineering, Institute of Technology of Cambodia, Phnom Penh, Cambodia

**Keywords:** Biogeochemistry, Environmental chemistry

## Abstract

To investigate the influence of chemical weathering on CO_2_ consumption, an analysis was performed of water chemistry by applying water chemistry equilibria methods in the Bishuiyan subterranean basin, SW China. The average value of total ion concentrations (TZ^+^) was 1,854.97 μEq/L, which was significantly higher than the global average value (TZ^+^ = 1,250 μEq/L). Ca^2+^ and HCO_3_^−^ were the main ionic constituents in the waters. SO_4_^2−^ and NO_3_^−^ concentrations were relatively higher than other anion concentrations, and Cl^−^ concentrations were consistently the lowest. Dissolved load balance models result showed that carbonate weathering, silicate weathering, and atmospheric input were the primary ionic contributors, wherein the effects of carbonate weathering > silicate weathering > atmospheric input for the whole catchment, with the exception of Taiping, where silicate weathering was prominent over carbonate weathering. In addition, these analyses indicated that the erosion via rock weathering was also affected by atmospherically derived CO_2_ and allogenic acids. The estimated yield by quantitative calculation for the carbonate weathering rate was 59.7 t/(km^2 ^year), which was 4.40 times higher than that of silicate weathering rate. Further, the carbonate and silicate weathering components of the carbon sink accounted for 71.2% and 28.8%, respectively, of the total basin rock weathering carbon sink.

## Introduction

Rock weathering in terrestrial ecosystems consumes atmospheric/soil carbon dioxide pools, thereby reducing the intensity of atmospheric greenhouse effects. Consequently, rock weathering is an important component to consider for geological carbon sinks^1–3^. Carbon sinks derived from carbonate weathering and silicate weathering are the two primary mechanisms underlying rock weathering carbon sinks^4^. Previous research on rock weathering has mainly focused on these processes in large river basins^5,6^. In these systems, the hydrochemical and isotopic compositions of waters are mainly controlled by the geology and lithology of basins^7^. Geochemical analyses of rivers can provide insight into chemical weathering within the basin, climate, and average chemical compositions and isotopic compositions in the upper crust, in addition to other important information for chemical elements involved in continental-river-ocean system allogenic cycling^8–10^. Rocks weathering progress and the levels of CO_2_ consumption have been analyzed in several large rivers, including the Congo^11^, Orinoco^12^ and Loire^13^ among others. Likewise, large river basins have been similarly investigated in China, including in the Yangtze^8,14^, Yellow^15,16^, Pearl^17,18^, Wujiang^7^, and Gan rivers^19^, in addition to Poyang Lake^20^ and others. These investigations have analyzed the chemical composition, ion runoff, chemical denudation, and climatic effects on waters, among other factors. Indeed, some studies have indicated that global river basin rock weathering is one of the primary components of global rock weathering, accounting for 87% of the carbon dioxide consumed in these processes^21^.

The CO_2_ consumptions of great river basins were influenced by many factors and thus difficult to evaluate^22,23^. The lithology, stratum structure, and vegetation conditions of small river basins are relatively simpler compared with lager basins, which makes it easier to investigate the influence factors of rock weathering in small basins^24^. Therefore, studying the chemical weathering of small watersheds can provide a more detailed comparison on the carbon sinks caused by chemical weathering. What’s more, it is of scientific interest to study the rock weathering in smaller watersheds where the recharge area is silicate rock and the downstream is carbonate rock. The consumption of atmospheric CO_2_ by the weathering of rocks with different lithological characteristics is different^21^. For example, Gaillardet et al.^21^ suggested that the carbon sink from global silicate weathering accounts for 40% of rock weathering flux, while the remainder arises from carbonate weathering. In contrast, Pokrovsky et al.^25^ suggested that the weathering rate of carbonate could be hundreds of times greater than that of silicate. Following this observation, the effects of carbonate weathering have been considered as underestimates^26,27^. Thus, it is critical to compare weathering rates and carbon sink effects arising from the weathering of carbonate and silicate in a typical watershed. To investigate these processes, the chemical composition of annual river runoff was analyzed in the subtropical granite/carbonate zone that is typical of the Guangxi Bishuiyan subterranean basin. Quantitative analyses of water chemical characteristics and the associated influencing factors were conducted by applying water chemistry equilibria methods. In addition, the rock weathering rates and the levels of atmospheric carbon dioxide absorbed during chemical weathering was evaluated. The overall aim of this study was to provide a baseline reference to investigate the influence of chemical weathering on carbon cycling.

## Materials and methods

### Study area

**Figure 1:**
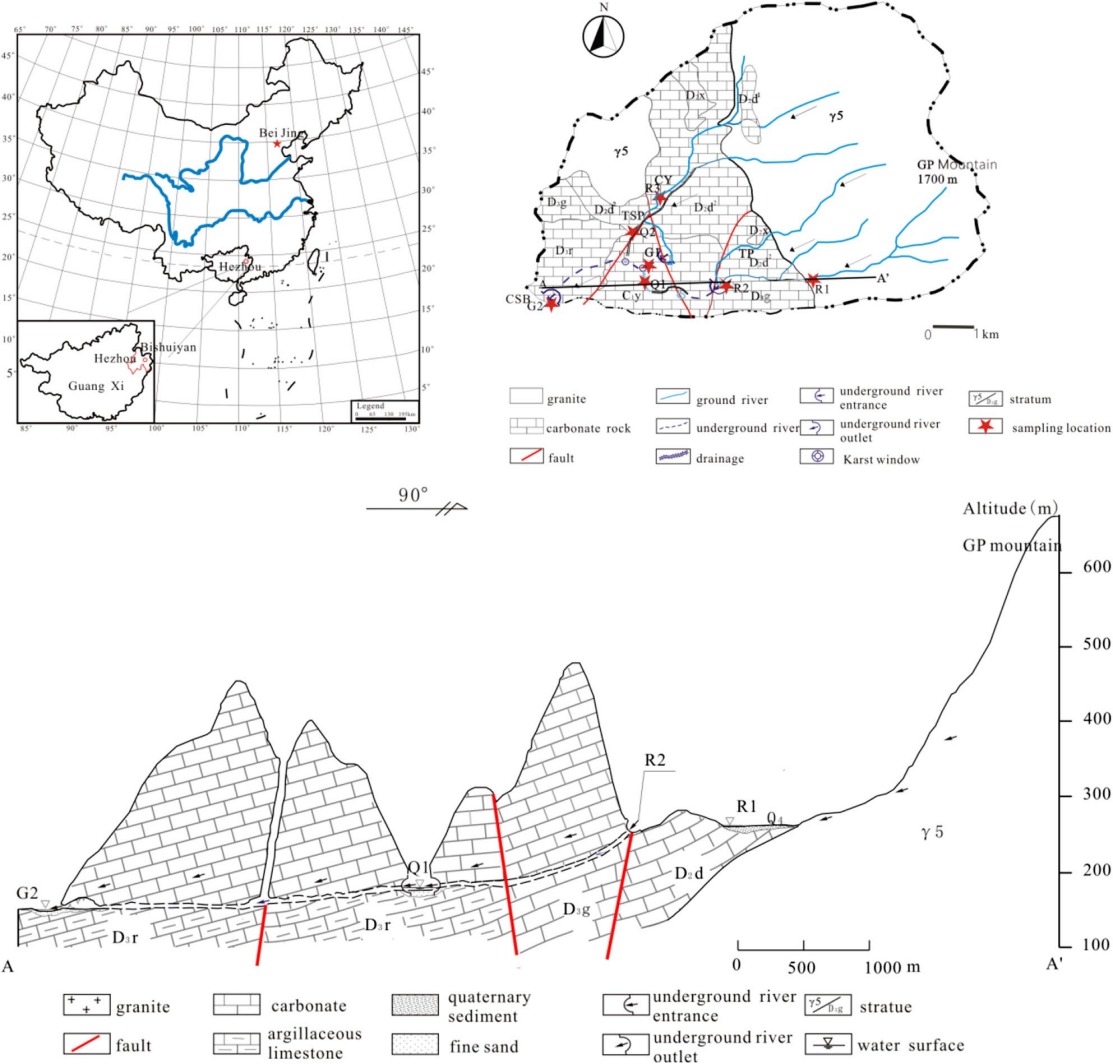
Map of the study area hydrogeology and an A–A′ geological profile section.

The Bishuiyan subterranean River is located in the town of Wanggao (E 111.448832–111.600838°, N 24.597314–24.652946°) within Hezhou City of the Guangxi Province, and it lies at the junction of Hunan, Guangdong, and Guangxi Provinces (Fig. 1). The geological structure of the basin comprises a granite body of GuPo (GP) Mountain and a contact zone of carbonate. The terrain exhibits high elevation in the east and low elevation in the west (Fig. 1). The lithology distribution in the area comprises multiple intrusive formations of granite complex rocks, where the central and western regions primarily consist of carbonate. The Bishuiyan River develops along the interface between a thick layered limestone of Devonian (D_3_r) age and thin argillaceous limestone, which comprises the main karst pipeline system of the area. The main channel of the river develops in the east–west direction. River water is mainly supplied by rainfall and surface runoff from granite formations. The granite watershed is the boundary to the north and east of the basin. However, the water acoustics groundwater system is relatively independent in the southern part of the basin. Geological structure and topography exert control on the river, resulting in a surface river that eventually flows into the Hejiang River. The upper stream of the underground river receives water supply from two granite water sources that transition to volcanic flows at Taiping (TP) and Tiejiaping (TSP), respectively. The river ultimately becomes a surficial system at the outlet wall, and runs as surface water for about 6 km. The underground river length comprises about 4.2 km. The flow rate at the outflow is 1,376–4,698 L/s, with the highest average flow rates from April to June, and the lowest rates from December to January.

The study area is adjacent to the Tropic of Cancer and features a subtropical monsoon climate with four distinct seasons. The average annual temperature of the area is 19.9 °C and the average annual rainfall is 1533.3 mm. Rainwater primarily occurs from April to August via large, episodic monsoon events that account for 60–80% of the annual rainfall of the area. Vegetation in the area is mainly forest that consists mostly of bushes. Agricultural activity is limited in the area and thus contributes little influence on the underground river.

### Hydrochemical parameters

Samples were collected from December 2014 to December 2015 at seven points along the river, including three in the upper stream (R1, R2, and R3), two at the drainage (Q1, Q2), one at the karst window (G1), and one at the underground river outlet (G2) (Fig. 1). Field measurements of pH, temperature, and electric conductivity (EC) were collected with a portable multi-parameter water quality analyzer (WTW multi 3,430, Germany). The analytical precisions for these measurements were 0.01, 1 μs/cm, and 0.1 °C, for pH, EC, and temperature, respectively. The pH and EC values were compensated to 25 °C.

### Analytical methods

Water samples were filtered using 0.45-μm acetate filter membranes and collected in 50-mL clear polyethylene bottles. Cations and anions were analyzed using two samples each. Cation samples were acidified (pH < 2) using 0.2 mL 1:1 HNO_3_, while the other water samples were preserved at 4 °C. All of the geochemical analyses were conducted at the Environmental and Geochemical Analysis Laboratory at the Institute of Karst Geology of the Chinese Academy of Geological Science. Cations (K^+^, Na^+^, Ca^2+^, and Mg^2+^) and anions (Cl^−^, SO_4_^2−^, and NO_3_^−^) were measured on an ICP-OES spectrometer (IRIS Intrepid II XSP, Thermo Fisher Scientific, USA) and Ion Chromatograph (861 Advanced Compact IC Metrohm, Swiss), respectively, with the analytical precision of 0.01 mg/L for both. HCO_3_^-^ was measured in triplicate via hydrochloric acid titration, and it exhibited an average error of less than 5%. SiO_2_ was measured based on the DZ/T0064.62-1993 method. Analytical precisions for HCO_3_^−^ and SiO_2_ analyses were both 0.1 mg/L.

### Calculation of atmospheric input and rock weathering

The Na ratio correction method was used to calculate equilibria values for water in order to quantify the contribution of three endmembers (carbonate weathering, silicate weathering, and atmospheric input) to the total carbon sink, based on previously described methods^8,16^. The method is also known as the Inversion Method and has been successfully applied to calculate equilibria chemistry for water of many global rivers^8,9,16,21,28^. The calculations are made by assuming that water solutes are the result of mixtures of different source materials, and that each material endmember exhibits different chemical characteristics. For each chemical element (Na, Ca, Mg, K, F, Cl, NO_3_, and Sr) or their isotopes, the following quantitative equilibrium equation can be applied:

1 $$ \left( {\frac{{\text{X}}}{{{\text{Na}}}}} \right)_{river} = \sum\limits_{i} {\left( {\frac{X}{{{\text{Na}}}}} \right)}_{i} \left( {\alpha_{{{\text{Na}}}} } \right)_{i} $$

where *i* represents different endmembers (i.e., atmospheric input, silicate weathering, and carbonate weathering), and (*α*_*Na*_)_*i*_ represents the quantity of Na among different solutes.

#### Atmospheric input

Human activity affects rock weathering in the Bishuiyan subterranean basin primarily through the emission of acid gases that fall via rainfall. The influence of these processes towards cation concentrations in the river basin is essentially negligible. Thus, balance of cations is primarily considered here (Na, K, Ca, and Mg) to calculate the contribution of the three endmembers to the cationic solutes of the river. Cation balance can be used to investigate the contribution of atmospheric inputs to water solutes via precipitation data and the water chemical composition within the Bishuiyan subterranean basin. Cl^−^ was used as the reference element to calculate the contribution of atmospheric precipitation to river chemistry, due to its conservative nature during water circulation^28,29^. The minimum Cl^−^ concentration of Bishuiyan subterranean basin waters was 12.4 μmol/L (G2) and 12.45 μmol/L in the Taiping branch of the upstream waters of Bishuiyan. The minimal difference between the two values can help distinguish the presence of accidental errors during water sampling. Consequently, the minimum Cl^−^ concentration (12.4 μmol/L) is considered to be entirely from atmospheric input. Using Formula (1)^28^, the content of other water components from atmospheric input can be assessed.

2 $$ {\text{X}}_{{_{{{\text{atm}}}} }}^{*} = \left( {X_{rain} \times {\text{Cl}}_{\min }^{ - } } \right)/{\text{Cl}}_{rain}^{ - } $$

#### Silicate weathering

Following the calculation of atmospheric input, the Na_sil_ composition in the water sample from silicate weathering can be calculated according to the following expression:

3 $$ \begin{aligned} \left[ {{\text{Na}}^{ + } } \right]_{{{\text{sil}}}} &= \left[ {{\text{Na}}^{ + } } \right]_{river} - \left[ {{\text{Na}}^{ + } } \right]_{atm} \\ & \approx \left[ {{\text{Na}}^{ + } } \right]_{river} - \left[ {{\text{Cl}}^{ - } } \right]_{atm} \\ \end{aligned} $$

4 $$ \left[ {{\text{K}}^{ + } } \right]_{{{\text{sil}}}} \approx \left[ {{\text{K}}^{ + } } \right]_{river} - \left[ {{\text{K}}^{ + } } \right]_{atm} $$

The ideal values of Ca^2+^ and Mg^2+^ from silicate weathering can be estimated from the chemical composition of the Taiping branch (R1). However, the powder industry (quarry stone processing) in the research area affects the content of Ca and Mg in some water samples. Consequently, the ratio of Ca^2+^/Na^+^ and Mg^2+^/Na^+^ from the river sample R1 was used that represented the granite background. The ratio refers to and modifies the corresponding value in the waters of the silicate basin in other research areas, with 0.55 and 0.25 as the values used to calculate silicate weathering in this paper^8,28^. [Ca]_sil_ and [Mg]_sil_ can be calculated by Eqs. (5) and (6).

5 $$ \left[ {{\text{Ca}}^{{{2} + }} } \right]_{{{\text{sil}}}} = \left[ {{\text{Na}}^{ + } } \right]_{sil} \times \left( {{\raise0.7ex\hbox{${{\text{Ca}}^{2 + } }$} \!\mathord{\left/ {\vphantom {{{\text{Ca}}^{2 + } } {{\text{Na}}^{ + } }}}\right.\kern-\nulldelimiterspace} \!\lower0.7ex\hbox{${{\text{Na}}^{ + } }$}}} \right)_{sil} $$

6 $$ \left[ {{\text{Mg}}^{{{2} + }} } \right]_{{{\text{sil}}}} = \left[ {{\text{Na}}^{ + } } \right]_{sil} \times \left( {{\raise0.7ex\hbox{${{\text{Mg}}^{2 + } }$} \!\mathord{\left/ {\vphantom {{{\text{Mg}}^{2 + } } {{\text{Na}}^{ + } }}}\right.\kern-\nulldelimiterspace} \!\lower0.7ex\hbox{${{\text{Na}}^{ + } }$}}} \right)_{sil} $$

Accordingly, the total amount of cations (TZ^+^) produced by silicate weathering and the corresponding contribution of solutes $\left( {\sum {{\text{Cation}}} } \right)_{sil}$ can be calculated as follows:

7 $$ {\text{TZ}}_{{{\text{sil}}}}^{ + } = 2 \times {\text{Ca}}_{{{\text{sil}}}}^{2 + } + 2 \times {\text{Mg}}_{{{\text{sil}}}}^{2 + } + {\text{Na}}_{{{\text{sil}}}}^{ + } + {\text{K}}_{{{\text{sil}}}}^{ + } $$

8 $$ \left( {\sum {{\text{Cation}}} } \right)_{sil} = {\raise0.7ex\hbox{${{\text{TZ}}_{{{\text{sil}}}}^{ + } }$} \!\mathord{\left/ {\vphantom {{{\text{TZ}}_{{{\text{sil}}}}^{ + } } {{\text{TZ}}^{ + } }}}\right.\kern-\nulldelimiterspace} \!\lower0.7ex\hbox{${{\text{TZ}}^{ + } }$}} \times 100{\text{\% }} $$

#### Carbonate weathering

Following analysis of Ca^2+^ and Mg^2+^ concentrations from atmospheric input and silicate rock weathering, the [Ca]_carb_ and [Mg]_carb_ from carbonate rock weathering can be calculated using Eqs. (8) and (9):

9 $$ {\text{Ca}}_{{{\text{carb}}}}^{2 + } = {\text{Ca}}_{{{\text{river}}}}^{2 + } - {\text{Ca}}_{{{\text{sil}}}}^{2 + } - {\text{Ca}}_{{{\text{atm}}}}^{2 + } $$

10 $$ {\text{Mg}}_{{{\text{carb}}}}^{2 + } = {\text{Mg}}_{{{\text{river}}}}^{2 + } - {\text{Mg}}_{{{\text{sil}}}}^{2 + } - {\text{Mg}}_{{{\text{carb}}}}^{2 + } $$

Correspondingly, the total concentration of cations (TZ^+^) from carbonate weathering and the solute contribution $\left( {\sum {{\text{Cation}}} } \right)_{sil}$ can be calculated as follows.

11 $$ {\text{TZ}}_{{{\text{carb}}}}^{ + } = 2 \times {\text{Ca}}_{{{\text{carb}}}}^{2 + } + 2 \times {\text{M}}g_{{{\text{carb}}}}^{2 + } $$

12 $$ \left( {\sum {{\text{Cation}}} } \right)_{{{\text{carb}}}} = {\raise0.7ex\hbox{${{\text{TZ}}_{{{\text{carb}}}}^{ + } }$} \!\mathord{\left/ {\vphantom {{{\text{TZ}}_{{{\text{carb}}}}^{ + } } {{\text{TZ}}^{ + } }}}\right.\kern-\nulldelimiterspace} \!\lower0.7ex\hbox{${{\text{TZ}}^{ + } }$}} \times 100{\text{\% }} $$

## Results and discussion

### Physicochemical parameters and total concentrations of dissolved ions

The pH of all of the water samples ranged from 6.68 to 8.33 (Table 1), indicating that the waters were circumneutral to alkaline. Conductivity values ranged from 21.1 to 331 μs/cm. The conductivity values of the R1 and R2 samples were relatively low (21.1–65.4 μs/cm), and reflected waters came from the granite host rock area. These values were also consistent with conductivity values measured upstream in the Zengjiang (42.7–66.9 μs/cm) and Pearl (27.2–78.6 μs/cm) Rivers^29^. The conductivity values of water samples in the carbonate area (G1, G2) and waters flowing through the carbonate zone (R3) were relatively higher (93.9–331 μs/cm). The result illustrated that the weathering rate of carbonate was higher than the weathering rate of silicate lead to the significant different of physicochemical parameters in samples^30^.

**Table 1:** Hydro-chemical measurements of water samples from the Bishuiyan subterranean basin.

	Measurement	Min	Max	Mean	S.E	C.V
Taiping tributary surface water (R1, R2)	Temp. (°C)	11.2	24.2	19.8	4.59	0.23
	pH	6.68	7.74	7.27	0.25	0.03
	EC (μs/cm)	21.1	65.4	41.8	12.3	0.29
	TDS (mg/L)	31.0	70.3	46.6	11.5	0.25
Chuanyan tributary surface water (R3)	Temp. (°C)	11.6	25.1	19.8	4.98	0.25
	pH	7.64	8.17	7.98	0.16	0.02
	EC (μs/cm)	93.9	331	248	70.1	0.28
	TDS (mg/L)	151	269	229	34.6	0.15
Taiping drainage water (Q1)	Temp. (°C)	13.6	24.8	20.8	3.78	0.18
	pH	7.08	8.01	7.70	0.28	0.04
	EC (μs/cm)	40.2	79.3	61.4	15.2	0.25
	TDS (mg/L)	41.1	72.2	57.6	11.3	0.20
Tieshiping drainage water (Q2)	Temp. (°C)	11.8	24.2	19.6	4.51	0.23
	pH	7.47	8.02	7.76	0.17	0.02
	EC (μs/cm)	78.4	270	138	69.0	0.50
	TDS (mg/L)	62.7	258	99.3	57.3	0.58
Swallet stream outlet (G1)	Temp. (°C)	18.0	21.7	20.1	1.24	0.06
	pH	7.51	7.85	7.66	0.11	0.01
	EC (μs/cm)	226	310	274	22.1	0.08
	TDS (mg/L)	217	268	248	14.6	0.06
Bishuiyan underground river Outlet (G2)	Temp. (°C)	13.8	23.8	19.8	3.29	0.17
	pH	7.42	8.33	7.70	0.26	0.03
	EC (μs/cm)	138	264	183	35.3	0.19
	TDS (mg/L)	127	242	158	33.6	0.21

TDS = [K^+^] + [Na^+^] + [Ca^2+^] + [Mg^2+^] + [Cl^−^] + [SO_4_^2−^] + [NO_3_^-^] + [HCO_3_^−^].

S.E., Standard error; C.V.: coefficient of variation.

In natural waters, the total number of cations (Ca^2+^, Mg^2+^, Na^+^, and K^+^) produced during mineral weathering is nearly equivalent to that of anions produced in aggressive medium^31,32^. The total cation concentrations of waters analyzed here ranged from 347 to 4,072 μEq/L, in which the result was similar to 60 rivers in the world (TZ^+^ = 300–10,000 μEq/L)^21^. The average value of TZ^+^ is 1855 μEq/L, which is higher than the global average value for rivers (1,250 μEq/L)^33^ and Qiantangjiang River (1357 μEq/L)^34^. The total anion concentrations of water samples ranged from 352–3,732 μEq/L, with an average value of 1803 μEq/L, which was significant higher than the Qiantangjiang River (1,363 μEq/L)^34^. Equilibrium coefficients (NIBC = (TZ^+^ − TZ^−^)/TZ^+^) ranged from − 9.97 to  + 9.80% with an average value of 1.26%. The typical range of NIBC values is − 10 to  + 10%.

### The spatial distribution of primary ionic components

Comparison of water chemical compositions from each cross section of the Bishuiyan subterranean basin indicated that upstream waters were significantly different from those downstream. Cation concentrations of R1, R2, and Q1 upstream waters exhibited trends of Ca^2+^ (0.11–0.31 mmol/L) > Na^+^ + K^+^ (0.07–0.15 mmol/L) > Mg^2+^ (0.01–0.08 mmol/L). The cationic composition was similar to that of Qiantangjiang River basin and Songhua River basin which were mainly composed of exposed silicate^9,34^. In contrast, the cation concentrations of G1, G2, R3, and Q2 waters followed trends of Ca^2+^ (0.31–1.45 mmol/L) > Mg^2+^ (0.10–0.64 mmol/L) > Na^+^ + K^+^ (0.07–0.19 mmol/L). This was similar to that of Wujiang River basin which was mainly composed of carbonate^35^. HCO_3_^−^ was the primary anion for all of the river waters, and accounted for 66.7%–95.0% of total anions. HCO_3_^−^ ranged from 0.30–0.63 mmol/L in R1, R2, and Q1 and 0.30–3.07 mmol/L for G1, G2, R3, and Q2. The other anions (in descending concentration) were NO_3_^−^, SO_4_^2−^, and Cl^−^. Ionic concentrations in upstream waters were significantly lower than that in the carbonate area, indicating that corrosion of carbonate considerably influenced the chemical properties of river waters.

### Qualitative analysis of ion sources

#### Chemical analysis of river waters

**Figure 2:**
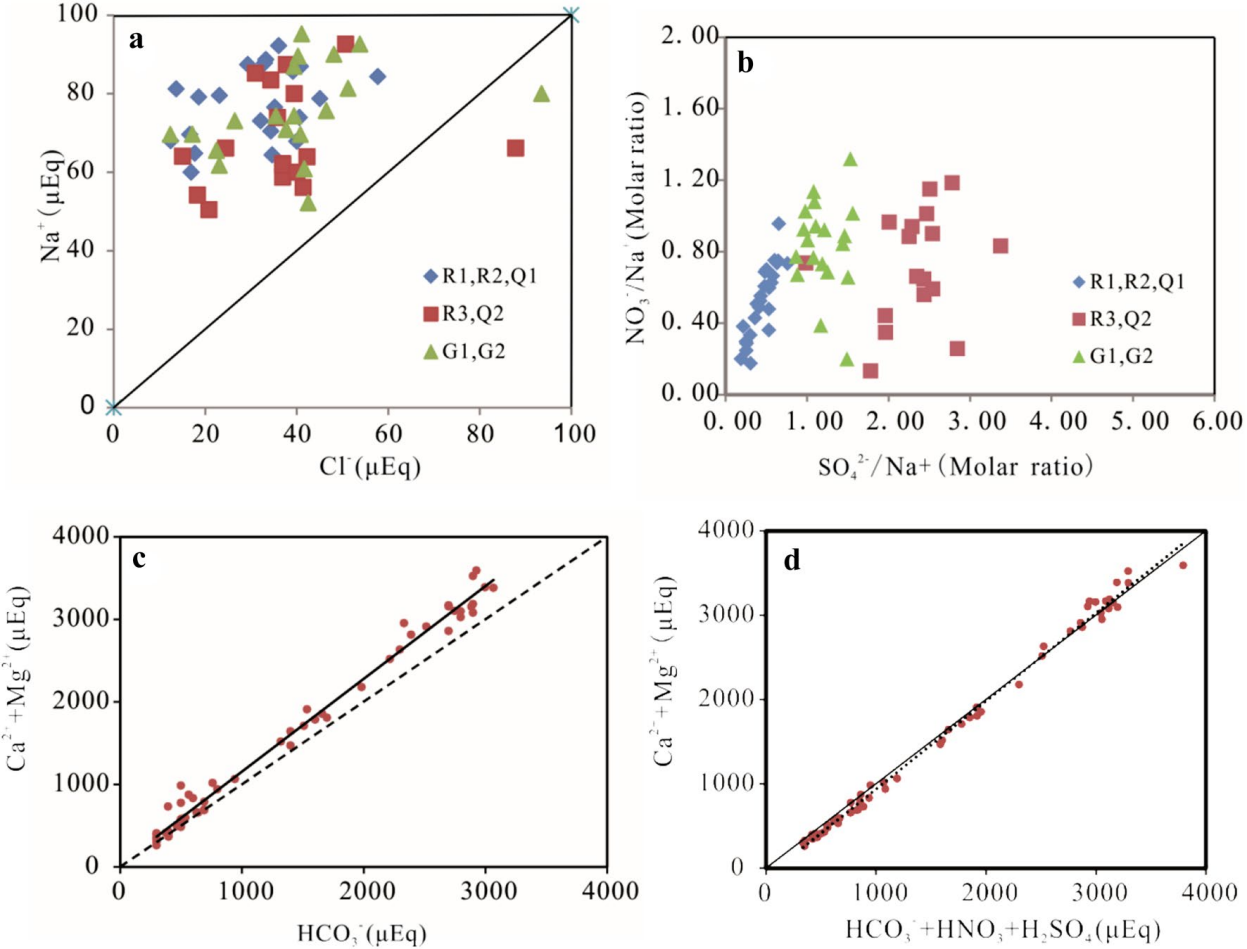
Relationships among major ions within waters of the Bishuiyan river basin.

Water chemical properties can reflect different sources or varying chemical conditions, as exhibited by particular elemental ratios^36^. Nearly all of the water samples fell above the equilibrium line of Na:Cl = 1 (Fig. 2a). These solute concentrations are influenced by marine aerosols, in addition to other factors^37^. In particular, the ratio of Ca^2+^ + Mg^2+^ and HCO_3_^-^ is typically used to identify carbonate weathering. The concentration of Ca^2+^ + Mg^2+^ was higher than that of HCO_3_^−^ in most of the samples (Fig. 2c). These results implicate the influence of acid from other sources in the weathering of carbonate^38^.

In addition to the erosive effect of H_2_CO_3_ derived from the atmospheric CO_2_, H_2_SO_4_ and HNO_3_ also make contributions to the rock weathering process (Fig. 2d). Previous studies showed that the chemical weathering by sulfuric acid played an important role in the chemical weathering of karst basin^39–41^. The sulfuric acid mainly come from atmospheric deposition, evaporate formation (gypsum/anhydrite and MgSO_4_) and oxidation of sulfides (pyrite)^37^. SO_4_^2−^ was positively correlated with NO_3_^−^ and Cl^−^ in Bishuiyan River waters, while SO_4_^2−^ was not obviously correlated with HCO_3_^−^. Further, SO_4_^2−^ and NO_3_^−^ were positively correlated with Na^+^ (Fig. 2b), indicating a similar source of SO_4_^2−^ and NO_3_^−^ as Cl^−^. Since there is no evaporates in the research area, the source of SO_4_^2−^ was not evaporates. It is likely that the allogenic acids in the river primarily derive from human activities and the oxidation of sulfides.

**Figure 3:**
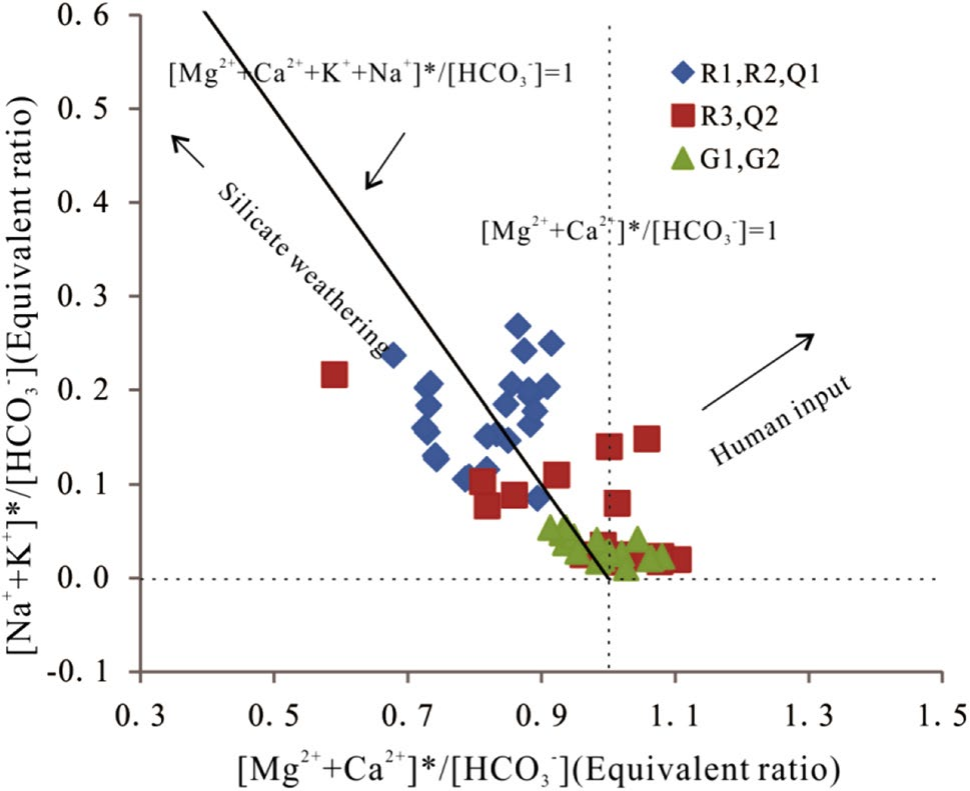
Relative contribution to water solute chemistry from silicate and carbonate weathering by carbonic acid.

Assuming that the allogenic acids (H_2_SO_4_ and HNO_3_) derived from human activities or sulfide oxidation were only used to balance Ca^2+^ and Mg^2+^ concentrations in the water, then [Ca^2+^ + Mg^2+^]*([Ca^2+^ + Mg^2+^]* = [Ca^2+^ + Mg^2+^] − [SO_4_^2−^ + NO_3_^−^]) originates from the weathering of carbonate and silicate. Therefore, the ratio of [Ca^2+^ + Mg^2+^]* to [HCO_3_^−^] represents the relative concentration of Ca^2+^ and Mg^2+^ from the weathering of carbonate and silicate, which should exhibit a ratio of less than 1.0. Similarly, the [Na^+^ + K^+^]*([Na^+^ + K^+^]* = [Na^+^ + K^+^] − [Cl^−^]) in the river results from the weathering of carbonate and silicate. Consequently, variation in the ratios of [Ca^2+^ + Mg^2+^]*/[HCO_3_^−^] and [Na^+^ + K^+^]*/[HCO_3_^−^] reflect the relative contribution of carbonate weathering and silicate weathering to solutes in the river water. The ratios for R1, R2, and Q1 waters fall on both sides of the 1:1 line, indicating that the water chemistry of the tributary water was influenced primarily by the weathering of silicate (Fig. 3). In contrast, water from the Chuanyan tributary and the exposed underground river in the carbonate area exhibited ratios of [Ca^2+^ + Mg^2+^]*/[HCO_3_^−^] = 1 and [Na^+^ + K^+^]*/[HCO_3_^−^] = 0, indicating that the water chemistry of the underground river was mainly controlled by the weathering of carbonate^42^. The [Ca^2+^ + Mg^2+^]* and [Na^+^ + K^+^]* values were higher than those for HCO_3_^-^ in the first quadrant of the graph, suggesting that excessive cations were not derived from the weathering of silicate and carbonate, but rather may be contributed by human activities. Consequently, it is likely that the anthropogenic contribution to cation concentrations was very small.

#### Identification of rock weathering source material

**Figure 4:**
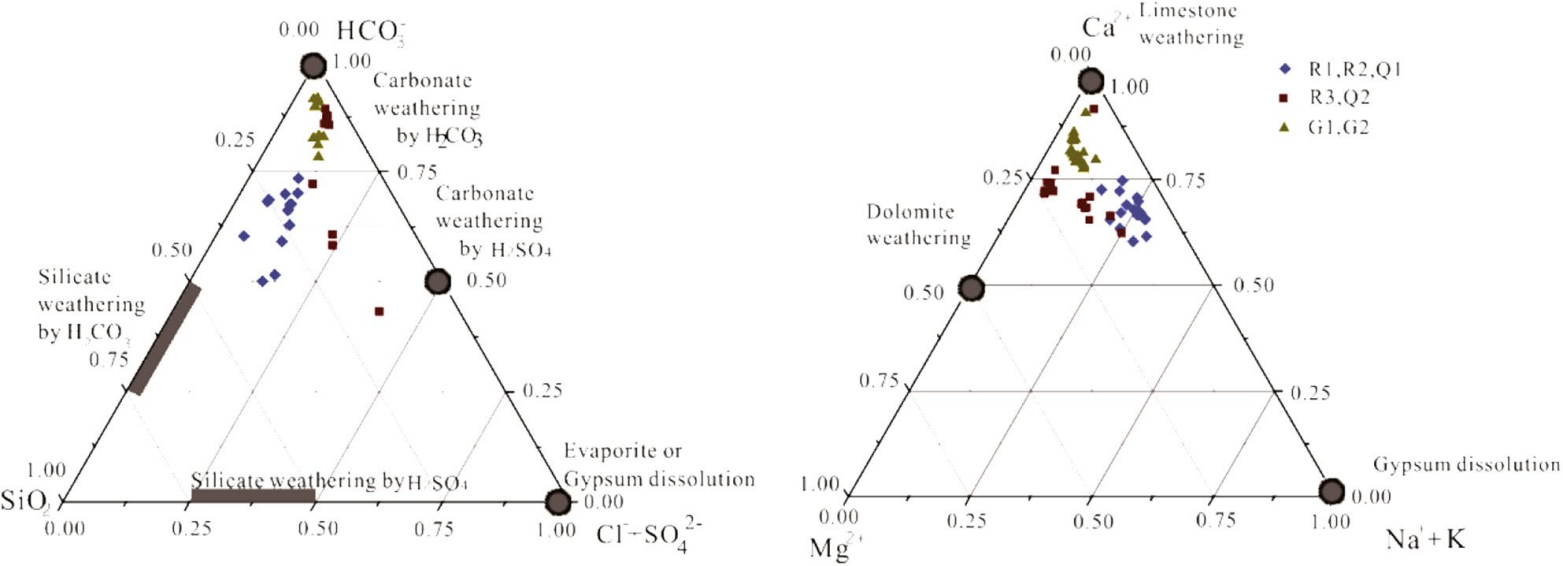
Triangle plots for major cations and anions of Bishuiyan river basin waters.

Triangular component compositional figures can aid analysis of water chemical data by aiding identification of water chemical compositions, the estimation of relative contributions of primary ions, and also help distinguish sources of solutes and their potential controls. Importantly, the relative contribution of chemical weathering of various rock minerals to dissolved solute loads of waters can be estimated through such analyses^43^. Triangular ionic compositional analysis of small rivers in the Bishuiyan subterranean basin (Fig. 4) indicated that cations were near the Ca^2+^ endmember at the exit of the Bishuiyan subterranean basin (G1, G2), while anions were reflective of an endmember water from carbonate weathering by H_2_CO_3_. The main cation in Taiping region water (R1, R2, and Q1) was Ca^2+^, and was also shifted towards the [Na^+^ + K^+^] endmember, while anions fell between the H_2_CO_3_-weathered carbonate and H_2_CO_3_-weathered silicate endmembers. The main cation of the Chuanyan region water (R3, Q2) was Ca^2+^, with a more minor contribution of Mg^2+^. However, the anion composition of these water was more atypical, reflecting the common influence from H_2_CO_3_-weathered carbonate in addition to H_2_CO_3_-weathered silicate and the H_2_SO_4_-weathered carbonate. These observations indicated that the solutes of the river water in the Bishuiyan basin were mainly controlled by carbonate weathering, silicate weathering, and atmospheric precipitation. Allogenic acids due to human activity also likely contributed from atmospheric precipitation. In addition, chemical weathering of the rock was primarily due to H_2_CO_3_-weathered carbonate, followed by H_2_CO_3_-weathered silicate. The effect of allogenic acids on rock weathering was mainly evident for carbonate, with little apparent effect on silicate.

### Quantitative estimation of water chemical constituents in the Bishuiyan river basin

#### Atmospheric input

The contributions of atmospheric inputs to different river sections of the Bishuiyan subterranean basin were calculated (Table 2). Cation components in the river water clearly varied among regions. Estimated atmospheric contribution rates to the R1, R2, R3, G1, and G2 water were 19.5–45.3% (average 33.2%), 19.7–35.9% (average 26.9%), 4.26–7.94% (average 5.26%), 4.53–5.58% (average 4.93%), and 4.94–10.1% (average 7.99%), respectively. The upper reaches were most affected by atmospheric inputs, while such influences were minimal in water in the carbonate area. In addition, the underground river was more resistant to contributions from atmospheric input compared to the surface river, as indicated by a smaller influence in ionic composition.

**Table 2:** Contributions from different inputs to cation contents in water samples from the Bishuiyan Basin.

		Atmospheric input					Silicate weathering					Carbonate weathering				
		R1	R2	R3	G1	G2	R1	R2	R3	G1	G2	R1	R2	R3	G1	G2
K (%)	Min	34.89	26.7	30.5	8.68	26.0	44.5	50.5	53.6	55.1	46.1	–	–	–	–	–
	Max	55.51	49.5	46.4	44.9	53.9	65.1	73.3	69.5	91.3	74.0	–	–	–	–	–
	Average	47.67	38.3	37.80	34.3	37.72	52.3	61.7	62.2	65.7	62.3	–	–	–	–	–
Na (%)	Min	6.12	5.34	7.05	6.26	5.92	90.6	91.3	88.8	89.2	90.7	–	–	–	–	–
	Max	9.40	8.70	11.18	10.81	9.26	93.9	94.7	93.0	93.7	94.1	–	–	–	–	–
	Average	7.73	6.77	9.34	7.76	7.56	92.3	93.2	90.7	92.2	92.4	–	–	–	–	–
Ca (%)	Min	26.3	23.1	5.55	4.94	4.95	21.6	19.1	2.97	2.32	2.81	0.48	23.8	85.2	90.0	82.0
	Max	66.0	46.2	11.0	6.30	12.0	42.6	38.2	4.94	4.79	8.58	56.7	61.9	92.1	93.2	92.8
	Average	44.4	35.3	7.20	5.60	9.37	30.1	27.8	3.99	3.91	6.67	32.0	42.8	89.7	91.3	85.4
Mg (%)	Min	4.34	5.47	0.56	1.05	1.30	29.4	24.6	2.80	4.86	9.02	1.36	9.66	95.4	89.7	86.8
	Max	21.1	14.9	1.13	1.67	2.39	118	86.5	4.22	10.4	13.7	71.1	70.8	97.0	94.7	90.9
	Average	12.6	9.77	0.74	1.22	1.97	68.9	52.1	3.37	6.95	11.3	32.3	35.8	96.4	93.0	88.6
TZ + (%)	Min	19.5	19.7	4.26	4.53	4.94	28.4	28.7	4.25	3.90	4.84	3.17	18.5	85.3	83.9	77.9
	Max	45.3	35.9	7.94	5.58	10.1	50.5	47.8	6.60	10.4	13.0	51.7	51.1	91.2	91.4	90.1
	Average	33.2	26.9	5.26	4.93	7.99	38.9	37.5	5.29	6.45	10.1	27.3	35.0	89.3	88.5	81.7

#### Silicate weathering

The average contributions of silicate weathering to the cation content of water samples in the research area were estimated for R1, R2, R3, G1, and G2 waters as 38.9%, 37.5%, 5.59%, 6.45%, and 10.1%, respectively (Table 2). The R1 sample was from water that were primarily granite-hosted. Hence, the influence of silicate weathering was significantly larger in R1, and the corresponding contribution change was larger than that of other river section water. This result was consistent with those described above, indicating that the weathering rate of silicate was greatly affected by seasonal changes.

#### Carbonate weathering

The average contribution of carbonate weathering to the cation content of R1, R2, R3, G1, and G2 samples were 27.3%, 35.0%, 89.3%, 88.5%, and 81.7%, respectively (Table 2). The results clearly indicated that during river runoff, increased contact with carbonate resulted in a gradual increase of carbonate components to the river water. Quantitative analysis also indicated that the water chemistry of the surface water or the underground river in the carbonate area was mainly controlled by the carbonate.

In summary, the analyses indicated differences in relative contributions of different endmembers to the solutes of different sections of the river. Silicate contributed most to the R1 and R2 water, followed by carbonate and then atmospheric input. Although there was only a small amount of carbonate in peripheral areas of R1, while the contributions of carbonate weathering and silicate weathering to the river solute were similar, due to the rapid dissolution^44^ or mixed dissolution^45,46^. The water chemistry of R1 and R2 may be typically controlled by silicate and carbonate weathering. In contrast, R3, G1, and G2 were primarily influenced by carbonate, silicate, and then atmospheric input. These results are consistent with the geological setting of the Bishuiyan subterranean basin, wherein water chemistry exhibits obvious regional characteristics. Lastly, the water chemistry of the Chuanyan branch water and the exposed underground river in the carbonate area (R3, G1, and G2), were mainly controlled by the weathering of carbonate.

### The chemical weathering rate of rocks in the Bishuiyan basin and the consumption of atmospheric CO_2_

The chemical weathering rate of rock minerals (t/(km^2 ^year)) is generally reflective of the embodiment of the weathering product of the rock minerals in the solutes of the river per unit area. Chemical weathering of carbonate and silicate was the primary control on the water chemical composition of the Bishuiyan subterranean basin. Relevant water chemistry and river flow data for the basin could then be used to calculate the weathering rate of silicate and carbonate, in addition to the consumption of atmospheric CO_2_, following previously described methods^9,47^ as indicated below.

Silicate weathering rate (SWR):

13 $$ {\text{SWR}} = {{\left( {\left[ {{\text{Na}}} \right]_{{{\text{sil}}}} + \left[ {\text{K}} \right]_{sil} + \left[ {{\text{Ca}}} \right]_{{{\text{sil}}}} + \left[ {{\text{Mg}}} \right]_{sil} + \left[ {{\text{SiO}}_{{2}} } \right]} \right) \times {\text{Q}}_{{{\text{annual}}}} } \mathord{\left/ {\vphantom {{\left( {\left[ {{\text{Na}}} \right]_{{{\text{sil}}}} + \left[ {\text{K}} \right]_{sil} + \left[ {{\text{Ca}}} \right]_{{{\text{sil}}}} + \left[ {{\text{Mg}}} \right]_{sil} + \left[ {{\text{SiO}}_{{2}} } \right]} \right) \times {\text{Q}}_{{{\text{annual}}}} } {\text{A}}}} \right. \kern-\nulldelimiterspace} {\text{A}}} $$

Carbonate weathering rate (CWR):

14 $$ {\text{CWR}} = {{\left( {\left[ {{\text{Ca}}} \right]_{{{\text{carb}}}} + \left[ {{\text{Mg}}} \right]_{{{\text{carb}}}} + 1/2\left[ {{\text{HCO}}_{{3}} } \right]_{{{\text{carb}}}}^{{}} } \right) \times {\text{Q}}_{{{\text{annual}}}} } \mathord{\left/ {\vphantom {{\left( {\left[ {{\text{Ca}}} \right]_{{{\text{carb}}}} + \left[ {{\text{Mg}}} \right]_{{{\text{carb}}}} + 1/2\left[ {{\text{HCO}}_{{3}} } \right]_{{{\text{carb}}}}^{{}} } \right) \times {\text{Q}}_{{{\text{annual}}}} } {\text{A}}}} \right. \kern-\nulldelimiterspace} {\text{A}}} $$

CO_2_ consumption rate during silicate and carbonate weathering:

15 $$ \phi {\text{CO}}_{{2}_{\text{sil}}} = ({\text{Na}}_{sil} +
{\text{K}}_{sil} + 2{\text{Mg}}_{sil} + 2{\text{Ca}}_{sil} ) \times
{\text{Q}}_{\text{annual}}/{\text{A}}$$

16 $$ \phi {\text{CO}}_{{2}_{{{\text{car}}}}} = {{({\text{Mg}}_{{{\text{car}}}} + {\text{Ca}}_{car} ) \times {\text{Q}}_{{{\text{annual}}}} } \mathord{\left/ {\vphantom {{({\text{Mg}}_{{{\text{car}}}} + {\text{Ca}}_{car} ) \times {\text{Q}}_{{{\text{annual}}}} } {\text{A}}}} \right. \kern-\nulldelimiterspace} {\text{A}}} $$

The cations produced by the weathering of carbonate and silicate can be calculated from Eqs. (3)–(10). To calculate the weathering rate of carbonate, the corresponding $\left[ {{\text{HCO}}_{{3}} } \right]_{{{\text{carb}}}}^{{}}$ value is first obtained. When the weathering rate of H_2_CO_3_-weathered carbonate and CO_2_ consumption are calculated, it is necessary to deduct the $\left[ {{\text{HCO}}_{{3}}^{ - } } \right]_{{{\text{carb}}}}^{{{\text{H}}_{2} {\text{SO}}_{4} + {\text{HNO}}_{3} }}$ released by allogenic acid due to [HCO_3_^−^]_carb_. If the ions are balanced in the process of silicate solution and erosion by H_2_CO_3_, then the following equation can be used:

17 $$ \left[ {{\text{HCO}}_{3}^{ - } } \right]_{{{\text{sil}}}} = {\text{CO}}_{{{2}}_{{{\text{sil}}}}} = \left[ {{\text{Na}}^{ + } } \right]_{{{\text{sil}}}} + \left[ {{\text{K}}^{ + } } \right]_{{{\text{sil}}}} + 2\left[ {{\text{Ca}}^{2 + } } \right]_{{{\text{sil}}}} + 2\left[ {{\text{Mg}}^{2 + } } \right]_{{{\text{sil}}}} $$

where the [HCO_3_^−^]_sil_ is the HCO_3_^−^ produced by silicate that is dissolved and eroded by H_2_CO_3_ in the water and CO_2sil_ refers to the atmospheric CO_2_ that is consumed in the process of dissolution and erosion.

To determine the [HCO_3_^−^]_carb_ produced during weathering of carbonate (including carbonic acid and allogenic acid dissolution and erosion), the following equation can be used:

18 $$ \begin{aligned}  \left[ {{\text{HCO}}_{{3}}^{ - } } \right]_{carb} &= \left[ {{\text{HCO}}_{{3}}^{ - } } \right]_{carb}^{{{\text{H}}_{2} {\text{CO}}_{3} }} + \left[ {{\text{HCO}}_{{3}}^{ - } } \right]_{carb}^{{{\text{H}}_{2} {\text{SO}}_{4} + {\text{HNO}}_{3} }} \\ &  = \left[ {{\text{HCO}}_{{3}}^{ - } } \right]_{total} - \left[ {{\text{HCO}}_{{3}}^{ - } } \right]_{sil} \\ \end{aligned} $$

where [HCO_3_^−^]_total_ is the total HCO_3_^−^ of the water; [HCO_3_^−^]_carb_ is the HCO_3_^-^ produced by dissolution and erosion of carbonate in the water; $\left[ {{\text{HCO}}_{{3}}^{ - } } \right]_{carb}^{{{\text{H}}_{2} {\text{CO}}_{3} }}$ is the HCO_3_^−^ produced by carbonate that are dissolved and eroded by H_2_CO_3_; and $\left[ {{\text{HCO}}_{{3}}^{ - } } \right]_{carb}^{{{\text{H}}_{2} {\text{SO}}_{4} + {\text{HNO}}_{3} }}$ is the HCO_3_^-^ produced by carbonate dissolution and erosion by allogenic acids (H_2_SO_4_ and HNO_3_) in the water.

The quantity of HCO_3_^−^ from various sources and the influence of various acids on the weathering of carbonate can be assessed via Eqs. (6) and (7). The weathering rate of H_2_CO_3_-weathered silicate, the weathering rate of carbonate eroded by carbonic acid and allogenic acids in the Bishuiyan subterranean basin, and the consumption of CO_2_ in corresponding process can be calculated using Formulas (2)–(8) (Table 3).

19 $$ \begin{aligned}  \left[ {{\text{HCO}}_{3}^{ - } } \right]_{{{\text{carb}}}}^{{{\text{H}}_{2} {\text{CO}}_{3} }} &= 2 \times \left[ {{\text{Ca}}^{2 + } + {\text{Mg}}^{2 + } } \right]_{{{\text{carb}}}}^{{{\text{H}}_{2} {\text{CO}}_{3} }} \\ & = 2 \times \left( {\left[ {{\text{HCO}}_{3}^{ - } } \right]_{{{\text{carb}}}} - \left[ {{\text{Ca}}^{{{2} + }} + {\text{Mg}}^{{{2} + }} } \right]_{{{\text{carb}}}} } \right) \\ \end{aligned} $$

**Table 3:** Weathering rates and CO_2_ consumption in the Bishuiyan subterranean basin waters.

Annual runoff	Silicate weathering		Carbonate weathering and allogenic acid weathering carbonate		Total	
Q, m^3^/year	SWR, t/(km^2^ year)	ØCO_2-sil_, 10^3^ mol/(km^2^ year)	CWR, t/(km^2^ year)	ØCO_2-carb_, 10^3^ mol/(km^2^ year)	WR, t/(km^2 ^year)	ØCO_2_, 10^3^ mol/(km^2^ year)
6.12 × 10^7^	13.6	192	59.7	476	733	668

The quantitative calculation of water chemistry resulted in an estimated rock weathering rate for the basin of 73.3 t/(km^2^ year), and an atmospheric CO_2_ consumption flux of 668 × 10^3^ mol/(km^2^ year), which are significant higher than the global rock weathering rate of 36 t/(km^2^ year) and the global atmospheric CO_2_ consumption flux of 246 × 10^3^ mol/(km^2^ year)^21^. The weathering rate and CO_2_ consumption flux in this study were slightly higher than the values in Yangtze basin, which were 85 t/(km^2^ year) and 611 × 10^3^ mol/(km^2^ year), respectively^21^. There are obvious climatic regional difference in the weathering rate and corresponding carbon sink capacity of the basin. For instance, Bishuiyan subterranean basin was subtropical monsoon climate, where the chemical weathering rate and atmospheric CO_2_ consumption flux were similar to the Pearl River basin and some tributaries of the Amazon basin (tropical rainforest climate)^18,48^. However, the corresponding values were significant lower than those in the Lesser Antilles (hot and humid climate, average annual temp. 24–28 ℃, average annual rainfall 2,400–4,600 mm), where the rock weathering rate and atmospheric CO_2_ consumption flux were (100–120 t/(km^2^ year)) and ((1,100–1,400) × 10^3^ mol/(km^2^ year)), respectively^49^. Meanwhile, the corresponding values in this study were lower than those in northern Okinawa Island (subtropical and humid climate, average annual temp. 22.2℃, average annual rainfall above 2000 mm) with the fluxes of CO_2_ consumed by silicate ((334–471) × 10^3^ mol/(km^2^ year))^50^. The rock weathering rate and atmospheric CO_2_ consumption flux of the basin located in the plateau climate and arid and semi-arid climate regions (low rainfall) were lower than those in hot and humid climate (high rainfall). The weathering rate and atmospheric CO_2_ consumption flux in Xinjiang rivers (average annual temp. 7–8 ℃, average annual rainfall 100–276 mm) were 0.12–93.6 t/(km^2^ year) and (0.19–284) × 10^3^ mol/(km^2^ year), respectively^29^. The weathering rate of rock and atmospheric CO_2_ consumption flux in the Songhua River basin (average annual temp. 3–5 ℃, average annual rainfall 500 mm) were (5.79 t/(km^2^ year)) and 190 × 10^3^ mol/(km^2^ year), respectively^9^. The atmospheric CO_2_ consumption flux in upper Yellow River in the Qinghai-Tibet Plateau (average annual temp. 1–8 ℃, average annual rainfall 434 mm) was 268 × 10^3^ mol/(km^2^ year)^51^.

Compared to the other small karst watersheds of in the similar climate, the atmospheric CO_2_ consumption flux in the study area was lower than that in Xiangxi Dalongdong underground river (819 × 10^3^ mol/(km^2^ year), average annual rainfall 1,800 mm) and four underground rivers in upstream of Wushui (878 × 10^3^ mol/(km^2^ year), average annual rainfall 1,444 mm). Meanwhile, the corresponding value was similar to that of Wanhuayan underground river (705 × 10^3^ mol/(km^2^ year), average annual rainfall 1565 mm)^52^. However, compared with the north karst of China^53,54^, the CO_2_ consumption flux in the study area was higher, resulting in the greater contribution to the rock weathering.

The comparison with other climatic zones in the world showed that the CO_2_ consumption caused by chemical weathering in the hot and humid climate zone is an important part of regulating atmospheric CO_2_ and constituting the global carbon balance. Besides, the small basins in karst (subtropical) area had relatively higher CO_2_ consumption. Therefore, the potential of chemical weathering carbon sink in some small subtropical basins with a wide distribution of carbonate is worth additional attention, which would provide some new insights for the scientific assessment of carbon sink effects caused by chemical weathering. Overall, the weathering of carbonate accounts for 71.2% (476 × 10^3^ mol/(km^2^ year)) of the carbon sink flux of weathered rocks in the Bishuiyan basin, while the weathering of silicate only accounts for 28.3% (192 × 10^3^ mol/(km^2^ year)). It indicates that more attention should be paid to the accurate assessment of the carbonate carbon sink intensity at the global and regional scales, the role and status of carbonate chemical weathering actively involved in the geological carbon cycle, is worth further study.

## Conclusions

A typical subtropical granite/carbonate zone was selected to analyze the chemical compositions of water representing annual river runoff. Moreover, rock weathering rates and atmospheric carbon dioxide absorption during chemical weathering were estimated. The average TZ^+^ of Bishuiyan subterranean basin water was 1855 μEq/L, which was above the global average for rivers (TZ^+^  = 1,250 μEq/L), while river water was mainly composed of Ca^2+^ and HCO_3_^−^. The conductivity of the river water (R1, R2) in the upstream tributary was relatively low (21.1–65.4 μs/cm), which is characteristic of water in granite settings. In contrast, the underground river water samples (G1, G2) were exposed to carbonate. Surface river (R3) water flowing through the carbonate zone had a relatively high conductivity, with values ranging from 93.9–331 μs/cm, suggesting a considerable influence from carbonate water–rock interactions.

Qualitative analysis of the ion sources in the river indicated the presence of additional allogenic acids from atmospheric deposition that was involved in the weathering and erosion of carbonates. The solutes in water of the Bishuiyan subterranean basin primarily derived from the weathering of carbonate, the weathering of silicate, and atmospheric inputs. However, in the upstream Taiping region of the Bishuiyan subterranean basin, the water chemistry was typically controlled by silicate and carbonate rock weathering. Lastly, the Chuanyan tributary and the exposed underground water in the carbonate area were mainly influenced by carbonate weathering inputs. This result showed that the small amount of carbonate has made almost the same contribution to solutes in the river water compared with the large amount of silicate. In addition, atmospheric CO_2_ and allogenic acids influenced rock weathering to a certain extent. Quantitative calculation of water chemistry suggested that carbonate weathering played an important role in the watershed carbon sink. The weathering rate of carbonate (59.7 t/(km^2^ year)) was 4.4 times higher than that of silicate (13.6 t/(km^2^ year)). The estimated carbon sink flux of carbonate chemical weathering was 2.4 times higher than that of silicate weathering. Compared with silicate rocks, the rapid chemical weathering rate of carbonate makes it worth further study in the evaluation of geological carbon sink.
